# ‘Fractional Recovery’ Analysis of a Presynaptic Synaptotagmin 1-Anchored Endocytic Protein Complex

**DOI:** 10.1371/journal.pone.0000067

**Published:** 2006-12-20

**Authors:** Rajesh Khanna, Qi Li, Elise F. Stanley

**Affiliations:** Toronto Western Research Institute, University Health Network Toronto, Ontario, Canada; University of Sydney, Australia

## Abstract

**Background:**

The integral synaptic vesicle protein and putative calcium sensor, synaptotagmin 1 (STG), has also been implicated in synaptic vesicle (SV) recovery. However, proteins with which STG interacts during SV endocytosis remain poorly understood. We have isolated an STG-associated endocytic complex (SAE) from presynaptic nerve terminals and have used a novel *fractional recovery* (*FR*) assay based on electrostatic dissociation to identify SAE components and map the complex structure. The location of SAE in the presynaptic terminal was determined by high-resolution quantitative immunocytochemistry at the chick ciliary ganglion giant calyx-type synapse.

**Methodology/Principle Findings:**

The first step in *FR* analysis was to immunoprecipitate (IP) the complex with an antibody against one protein component (the IP-protein). The immobilized complex was then exposed to a high salt (1150 mM) stress-test that caused shedding of co-immunoprecipitated proteins (co-IP-proteins). A *Fractional Recovery* ratio (*FR*: recovery after high salt/recovery with control salt as assayed by Western blot) was calculated for each co-IP-protein. These *FR* values reflect complex structure since an easily dissociated protein, with a low *FR* value, cannot be intermediary between the IP-protein and a salt-resistant protein. The structure of the complex was mapped and a blueprint generated with a pair of *FR* analyses generated using two different IP-proteins. The blueprint of SAE contains an AP180/*X*/STG/stonin 2/intersectin/epsin core (*X* is unknown and epsin is hypothesized), and an AP2 adaptor, H-/L-clathrin coat and dynamin scission protein perimeter. Quantitative immunocytochemistry (ICA/ICQ method) at an isolated calyx-type presynaptic terminal indicates that this complex is associated with STG at the presynaptic transmitter release face but not with STG on intracellular synaptic vesicles.

**Conclusions/Significance:**

We hypothesize that the SAE serves as a recognition site and also as a seed complex for clathrin-mediated synaptic vesicle recovery. The combination of *FR* analysis with quantitative immunocytochemistry provides a novel and effective strategy for the identification and characterization of biologically-relevant multi-molecular complexes.

## Introduction

Transmitter release is triggered by the influx of calcium ions through local calcium channels[1] which bind to a calcium sensor on the transmitter release site and gate the fusion of the docked synaptic vesicles (SV) with the surface membrane. After discharge of the vesicle contents, the SV is recovered by a clathrin dependent endocytosis mechanism[2]–[4]. Coat formation, cargo capture, vesicle budding and dynamin-dependent scission involve a large number of proteins and protein interactions (see[5], [6] for review). Synaptotagmin 1 (STG) is best known as the putative calcium ion sensor in the triggering of SV fusion. Interestingly, however, this protein has also been implicated as a regulator of endocytosis and interference with STG markedly slows the rate of vesicle recovery[7]–[11]. Recent observations demonstrate that STG remains clustered even after the secretory vesicle has fused,[12] and suggests that the protein is a component of a relatively stable molecular complex associated with endocytosis. Our objective was to analyze this STG-endocytosis-related complex.

Our first observation was the STG co-precipitates from purified synaptosome lysate with a number of endocytosis related proteins, including the clathrin coat proteins, heavy and light chain clathrin (H-clathrin; L-clathrin) and dynamin, adaptor protein such as AP2 and also linker proteins such as intersectin and AP180. We term this entity an STG-associated endocytic (SAE) complex. In order to test which of the SAE protein components was more closely associated with STG we explored these linkages using a high-salt stress test, determining *Fractional Recovery* (*FR*) values for each protein link. This experimental strategy is simple and yet compelling: proteins that are readily shed with high salt, (low *FR*) can not serve as intermediates between the IP-protein and proteins that remain bound (high *FR*). Thus, the rank order of the *FR* values, the *FR* *sequence*, provides a clue to the interrelationship between the individual members of a multi-molecular complex. We reasoned that we could gain further insight into the substructure of a multi-molecular complex by deriving the *FR* sequence based on a second IP-protein from the same complex. We then used these *two FR* sequences as coordinates to fix the location of each protein in the complex.

The SAE was examined using four different *FR* sequence pairs and the resulting complex structures were compared to derive a consensus model. Some of the proteins that abut in this model were previously described binding partners but others were not. We hypothesized bridging proteins by a search of the literature for proteins known to bind the two proteins on either side of each gap. We then carried out additional *FR* experiments to test putative bridging proteins.

Our analysis generated a blueprint of a novel multi-protein complex with an endocytosis adaptor-protein core and coat-protein periphery. Quantitative immunocytochemistry (ICA/ICQ method, [13]) was used to determine the sub-cellular location of the complex within a native presynaptic terminal. The SAE was localized the to the transmitter release face region, consistent with a role in secretory vesicle recycling.

## Results

### Synaptotagmin co-precipitates with endocytotic proteins from purified synaptosome lysate

**Figure 1:**
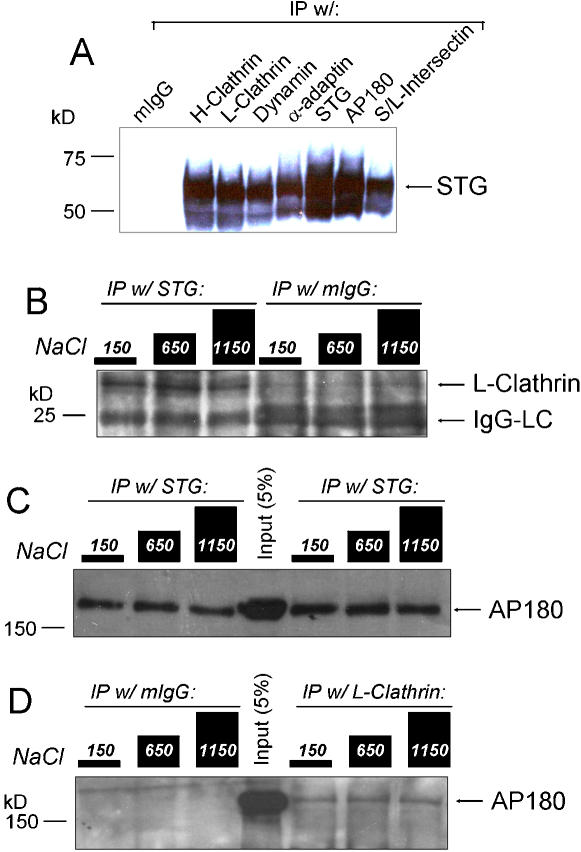
Fractional recovery method. ***A.*** Rat brain synaptosome lysate was immunoprecipitated with an antibody against the protein indicated above the lanes (the IP-protein). The captured proteins were eluted off the beads and were identified by standard Western blot analysis. Synaptotagmin 1 (STG) was co-precipitated with all of the endocytosis proteins tested. S-/L-intersectin: short/long-variant-intersectin. ***B.–D.*** After immunoprecipitation as in ***A***, the beads were washed and split into three equal aliquots. Each aliquot was exposed to one of three buffers with different salt concentrations before elution of the remaining bead-attached proteins and analysis by Western blot. In each set of three lanes the left lane was exposed to control 150 mM salt, the center lane to 650 mM and the right lane to 1150 mM salt, as indicated by the diagram at the top. ***B.*** Immunoprecipitation of synaptotagmin co-immunoprecipitated L-clathrin (***left three lanes***) with only a small reduction at high salt whereas IgG was negative (***right three lanes***). ***C.*** The fractional recovery method was repeatable. In this panel AP180 was co-precipitated with STG in two separate trials (***left three and right three lanes***) with similar salt-resistant results. ***D.*** ***Figures 1B and 1C*** show co-precipitation of L-clathrin with STG and AP180 with L-clathrin and both of these are resistant to high salt treatment. Consistent with these findings, AP180 co-precipitates with L-clathrin (***right three lanes***) in a salt-resistant manner whereas IP with IgG is negative (***left three lanes***). In ***C*** and ***D*** the ***center lane*** is 5% of the loading lysate used for the immunoprecipitation.

**Figure 2:**
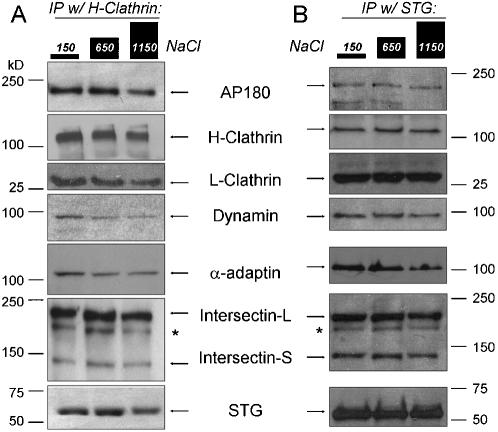
Endocytosis-related proteins co-precipitate from rat brain synaptosomes and co-precipitated protein recovery is ionic strengthsensitive. Proteins immunoprecipitated with H-clathrin (***left panel***) or STG (***right panel***) from rat synaptosome lysate after exposure to the three salt buffers. Each panel shows bands that correspond in molecular weight to the labeled protein recovered after the three salt treatments (see ***Fig. 1*** legend). For example, comparing the 1150 mM salt-treated to the control, 150 mM salt-treated, there was a high recovery of H-clathrin, a moderate recovery of L-clathrin or S-intersectin and a low recovery of dynamin. The asterisk denotes an intermediate intersectin proteolytic fragment, as reported previously.[31] Other details as in ***Figure 1***.

Synaptosomes were prepared from 2–10 day rats and were solubilized in a standard detergent-containing lysis buffer at a physiological ionic strength of 150 mM. We used standard co-immunoprecipitation to test for an STG endocytosis complex. Immunoprecipitation of a range of endocytosis-related proteins, including adaptors such as AP180, intersectin and AP2 (α-adaptin), the coat proteins H- and L-clathrin and the scission protein dynamin co-precipitate with STG (***Fig. 1A*** ***,*** ***2B***). However, immunoprecipitation alone gives little information with respect to the strength of these associations or the proximity of each IP-protein to STG, the co-IP protein. In order to obtain more information we stressed the electrostatic interactions within the complex by exposing it to buffers containing elevated salt. We tested two salt concentrations, 650 and 1150 mM, and compared these to a 150 mM control. Exposure of the immobilized complex to 1150 mM salt for 15 minutes was selected as an effective and experimentally repeatable (***Fig. 1B–D***) assay protocol that liberated a significant fraction of the protein members (***Fig. 2*** ***,*** ***3***). The 650 mM salt served as a control for non-specific or very weakly bound components: only proteins with >40% mean retention at this ionic strength were included in the complex. Thus, each immunoprecipitation experiment was split into three identical samples which were treated for 15 minutes in 150, 650 and 1150 mM salt. Proteins that remained bound to the precipitation beads were eluted by boiling in a denaturing buffer and were assayed by immunoblotting and densitometry (see ***Supporting Information Fig. S1A, B***). A *fractional recovery* (*FR*) value was defined as the ratio of protein recovered after exposure to the highest salt (1150 mM) concentration divided by that at 150 mM. Not counting controls, data from over 240 individual immunoprecipitation experiments, each employing a three-salt paradigm, were carried out in this study.

**Figure 3:**
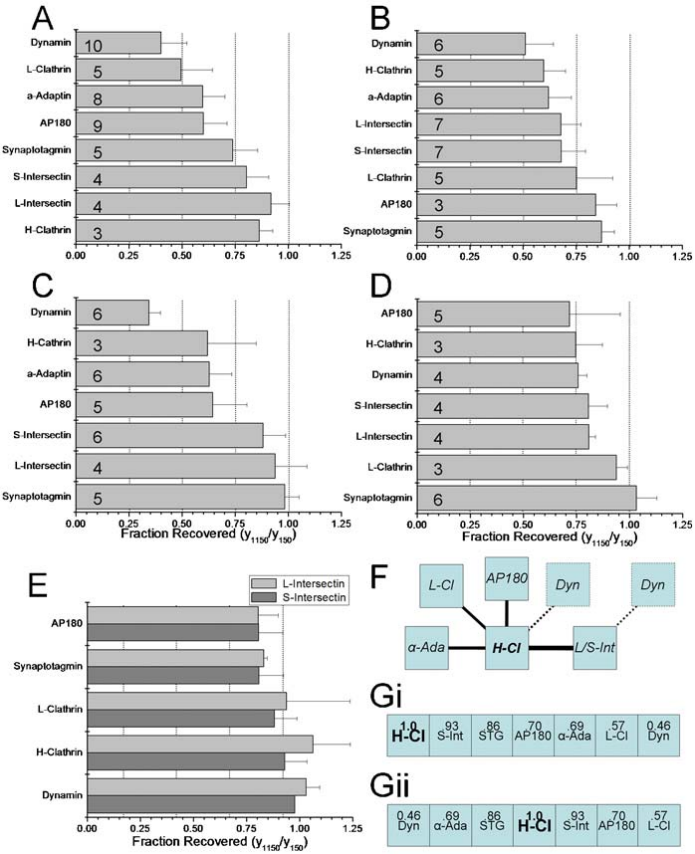
FR analysis of SAE. *Analysis frequency histograms.* Each plot shows the fraction of a variety of co-IP-protein recovered at 1150∶150 mM salt after immunoprecipitation with antibodies to (***A***) H-clathrin, (***B***) AP180, (***C***) L-clathrin, and (***D***) STG (the IP-proteins). Each bar is the mean±SE of the indicated number of separate IP experiments as in ***Fig. 1C***. The vertical dotted lines indicate the 0.25, 0.50, 0.75 and 1.00 recovery fractions. ***E.*** Plot of mean±SE *FR* values for IP of S- or L-intersectin compiled from co-IP values for the indicated IP-proteins (*y axis*; *see text*). ***F, G.*** *Protein complex model relative to the IP-protein*. Working models of the protein complex depicted by the FR set for a single IP-protein using H-clathrin as an example. In ***F*** the co-IP proteins are displayed with linking bars with widths proportional to their *FR* value (there is no significance to the distance between the proteins in this diagram). Dynamin is depicted as either linked directly to H-clathrin or, because of its low *FR* value, linked via intersectin (*see text*). In ***G*** the complex is depicted as a linear sequence of co-IP proteins in order of their normalized *FR* values (number in box) starting from the IP protein, H-clathrin, on one end (***i***) or at the centre (***ii***) of the complex. *FR* values for S- and L-intersectin were very similar as if they can substitute for each other in this complex. Figure abbreviations: S-Int, short-variant intersectin; STG, synaptotagmin; α-Ada, α-adaptin; L-Cl, L-clathrin; Dyn, dynamin.

***Figure 1B-D*** illustrates the method. STG (the IP-protein) was immunoprecipitated from the lysate and we probed for L-clathrin (a co-IP-protein). L-clathrin bands were detected in the Western blot after all three salt treatments (***Fig. 1B***). This method was found to be repeatable (e.g. ***Fig. 1C***) and precipitations were reciprocal (***Figs 1B–D*** ***, Supporting Information, Figure S1***) while IgG controls were negative or very faint (***Figs 1B, D***).

Immunoprecipitation of H-clathrin or STG both captured a range of endocytosis-related proteins (***Fig. 2A, B*** ***; left lanes***). These findings support the presence in the lysate of a multi-protein complex, the SAE complex, but yield little information as to its molecular organization. However, we argue that the sequence of co-IP-protein *FR* values with a given IP-protein is an indicator of the internal structure of the complex relative to that IP-protein (*see below*).

### FR values

We carried out a full set of salt sensitivity experiments on four immunoprecipitated complex components: STG, H-clathrin (***Fig. 2A, B***), AP180 and L-clathrin (*data not shown*,) together with limited analyses with dynamin and α-adaptin. In each case we first tested the *FR* value of the IP-protein itself in order to ensure that the high salt did not simply liberate the antibody from the bead or the IP-protein from the antibody. >75% recovery was obtained in all four cases (***Fig. 3A–D***). The *FR* values for each of the co-IP-proteins (***Fig. 2A–D***) were normalized to that for the IP-protein. Thus, we generated sets of FR values for each IP-protein for which we had a reliable antibody. We did not have antibodies that can distinguish between the two S- and L- variants of intersectin, precluding the experimental determination of their respective *FR* value sets. However, *FR* values were reciprocal, that is the *FR* value of co-IP-protein *A* with IP-protein *B* was in essence the same as that for co-IP-protein *B* with IP-protein *A* (***Fig. 1B–D***; ***SupportingFig. S1C***). Thus, we generated FR value sets for the two intersectin variants by compiling their respective *FR* values using the other IP-proteins (***Fig. 3E***).

### Analysis of complex organization by FR values

#### i. FR values reflect sequence of proteins in the complex

The diagram in ***Fig. 3F*** depicts the degree to which high salt displaced the members of the protein complex from the IP-protein, in this case H-clathrin. This diagram is purely descriptive, and has only limited information with respect to the structure of the parent complex. The *FR* values can, however, be used to predict favorable linear protein series within the complex because *a co-IP-protein that dissociates readily in high salt can not also serve as the key bridge between the IP-protein and a more salt-resistant second co-IP-protein*. Thus, in ***Fig. 3F*** since dynamin has the lowest *FR* value, it could be sited attached to H-clathrin or beyond any of the other co-IP proteins. However, its most likely location is attached to a known binding partner such as intersectin.[14]

Since many complexes involve linear protein arrays, an alternative and, as it turns out, more useful working model is to arrange the co-IP-proteins in a linear sequence according to their *FR* values (***Fig. 3G***). However, this arrangement can not distinguish a structural model in which the IP-protein is at one end of the complex with the co-IP-proteins arranged in a linear series (***Fig. 3Gi***), from a more complex models where the IP-protein is in the middle and the co-IP-proteins radiate in two or more directions (e.g. ***Fig. 3Gii***). Further, it can not detect branches within the structure. The creation of a more useful organizational model of the complex requires more data.

#### ii. Protein complex analysis using multiple IP-protein FR sequences

We rationalized that a blueprint of the complex might be derived by a comparison of two *FR* sequences obtained with two different IP-proteins. It was first necessary, however, to ensure that these sequences reflect the same protein complex within the tissue lysate. This assumption was supported by the finding that the *FR* values were reciprocal (***Supporting Information Fig. S1C***, see also discussion above). While it is undoubtedly the case that the proteins associated with the SAE complex are also components of other key presynaptic terminal lysate complexes, the SAE appears to be common so that it dominates the *FR* analysis. We also devised a test for complex congruence with different IP-proteins (***Supporting Information Fig. S2***). In essence, this tests if the *FR* value sets generated by pairs of IP-proteins originate from the same complex. The finding that all the protein pairs generated highly congruent *FR* value sets suggests that the SAE must be the predominant endocytosis complex in our lysate.

**Figure 4:**
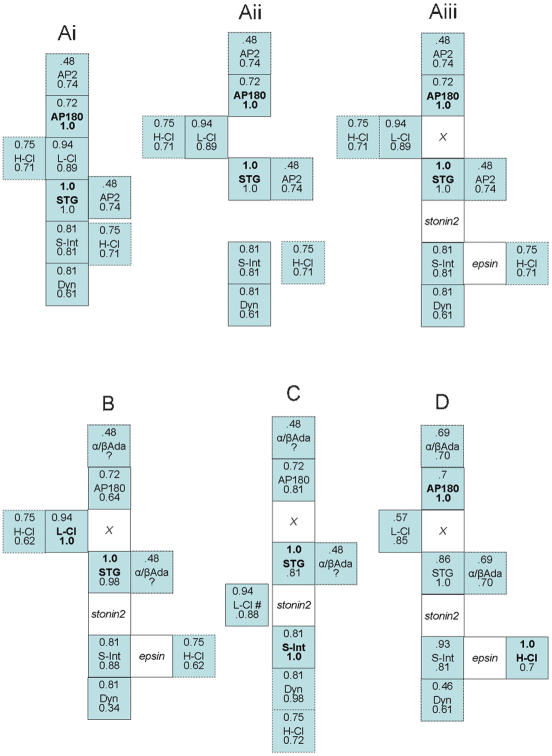
FR protein complex analysis. A. Each figure is a model of the protein complex with each protein indicated by a box together with their respective IP-protein *FR* values (see ***Fig 3A***). Thus, in ***Ai–iii*** AP180 is one IP-protein and the upper numbers for each co-IP-protein reflect its *FR* values. Similarly, STG is the second IP-protein and the lower numbers in each box are its *FR* values. The *FR* value for the IP-protein itself is always 1.0. Proteins are mapped to a single locus (solid line box), two or three loci (dashed line box) or to multiple loci (dotted line) with only a few possible positions shown. Hypothetical proteins are indicated in italics. ***Ai–iii***. Analysis method (see text). ***B–D***. Complex structure as predicted by three additional IP-protein pairs: STG/L-Cl (***B***); STG/S-Int (***C***) and AP180/H-Cl (***D***). Note all four bait pairs generate models with similar cores while most of the variations occur in the extremities. STG, synaptotagmin; L-Cl, L-clathrin; H-Cl, H-clathrin; stonin, stonin2; *X* unknown protein or protein complex. Abbreviations are as in ***Fig. 2***.

An example of the analysis of protein complex organization using interacting *FR* series is presented in ***Fig. 4Ai–iii*** using AP180 and STG as the IP-proteins. First, these two IP-proteins were given a provisional location relative to each other. Generally, if the reciprocal *FR* values are very different, the two IP-proteins are probably distant within the complex, and *vice versa*. The co-IP-proteins were then added one at a time, using their corresponding *FR* values as coordinates. Typically, a pair of high *FR* values indicates a location between the IP-proteins while one high and one low value indicate a location distal to one or other IP-protein (***Fig. 4Ai***). Thus, L-clathrin was located between AP180 and STG. Dynamin was distal to STG as it has a high STG but a low AP180 *FR* value and fell distal to intersectin. H-clathrin had low *FR* values for both STG and AP180 and could be located at any of three positions (for simplicity the third location, attached to STG, is not shown). The α-adaptin-containing AP2 complex is highly promiscuous and due to its low *FR* values could be positioned at several attachment sites.

The above analysis results in a map of proteins within the SAE complex. Some of these proteins (e.g. dynamin and intersectin) are known binding partners whereas others are not (e.g. AP180 and STG). Thus, the next step (***Fig. 4Aii***) was to incorporate previously reported (***abutting boxes***) and unknown binding interactions (***gaps***) into the model. We searched the literature and on-line binding databases (BIND, PPID) to identify proteins that might bridge the gaps (***Fig. 4Aiii***). For simplicity, we limited the search to a single bridging protein in each case. Thus, the gap between STG and intersectin could be bridged by stonin2.[15], [16] A possible direct link between intersectin [17] and H-clathrin[18] is offered by epsin.[16] We were unable, however, to span the AP180/STG/L-Cl gap (labeled ***‘X’***) by a single protein and suspect a multi-protein or novel link. While AP2 was a potential candidate, the low α-adaptin *FR* rules out this option.

We repeated the steps above using the *FR* sequences for three more IP-protein pairs using the data in (***Fig. 3A–E***). The resulting organizational models exhibited strong similarities to that presented in ***Fig. 4Aiii***, in particular with respect to the complex core (defined as the proteins with more than one attached partner).

### Model prediction

**Figure 5:**
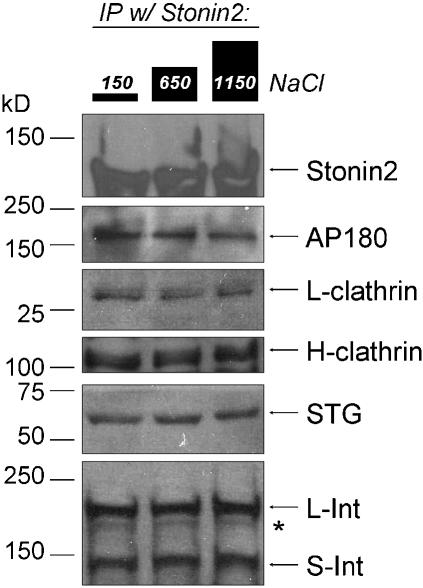
Effect of high salt on recovery of proteins co-precipitated with stonin2. Legend as in ***Figure 1C***.

**Figure 6:**
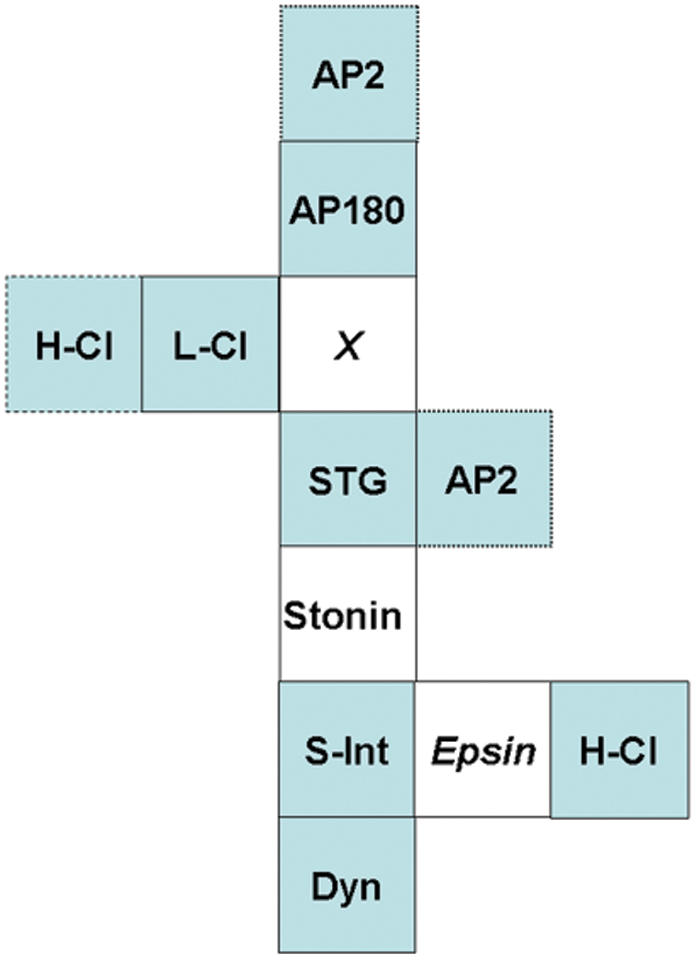
Consensus model for SAE. Model based on Fig. 3 ***Aiii, B, C*** and ***D*** with stonin2. H-Clathrin is most likely located abutting eprin but could also be attached to L-Clathrin. Note that L-intersectin was very similar to S-intersectin throughout. We assume that α-adaptin is marker of the entire AP2 adaptor complex. Abbreviations and notations as in ***Fig. 3*** but without *FR* values.

The proteins that are predicted in the above analyses to span gaps provide a demanding test for the validity of the *FR* method. The very recent report of an antibody against stonin2[19] gave us a unique opportunity to carry out such a test. We predicted that: stonin2 co-immunoprecipitates with the entire protein set and, a more stringent test, that its *FR* values would be consistent with a location between STG and intersectin. Thus, from ***Fig. 4Aiii, B–D***, stonin2 should have an *FR* value greater than 0.81 relative to AP180 or STG; a value between 0.88 and 0.98 relative to L-clathrin; greater than 0.81 for S- or L-Intersectin, and between 0.86 and 0.93 for H-clathrin. Stonin2 co-immunoprecipitated with all complex proteins tested (***Fig. 5***). Since immunoprecipitated stonin2 was retained on the bead in high salt (stonin 2: *FR* = 0.94±0.08, N = 6) it was possible to use this antibody for a full *FR* analysis. Experimentally determined *FR* means were: STG, 0.98±0.07 (N = 5); L-clathrin, 0.84±0.16 (N = 5); AP180, 1.00±0.17; L-intersectin, 1.0±0.04 (N = 3); S-intersectin, 0.83±0.18 (N = 3); and H-clathrin, 0.78±0.11 (N = 6). These values were obtained with stonin2 as the IP-protein except for AP180 and H-clathrin where these proteins were IP-proteins while immunoblotting for stonin2. Our findings demonstrate that the predicted and experimentally determined *FR* values were remarkably similar and support the hypothesis that stonin2 links STG and intersectin as in ***Fig. 4Aiii. B. C. and D***. We can compile these models for a consensus SAE blueprint (***Fig. 6***). The lack of a suitable antibody prevented us from testing epsin as the second putative bridge. Thus, our model of SAE has an AP180-*X*-STG-stonin 2-intersectin-dynamin core with one preferred and a second likely location for H-clathrin, and at least two possible locations for AP2.

### Localization of SAE at the presynaptic terminal

**Figure 7:**
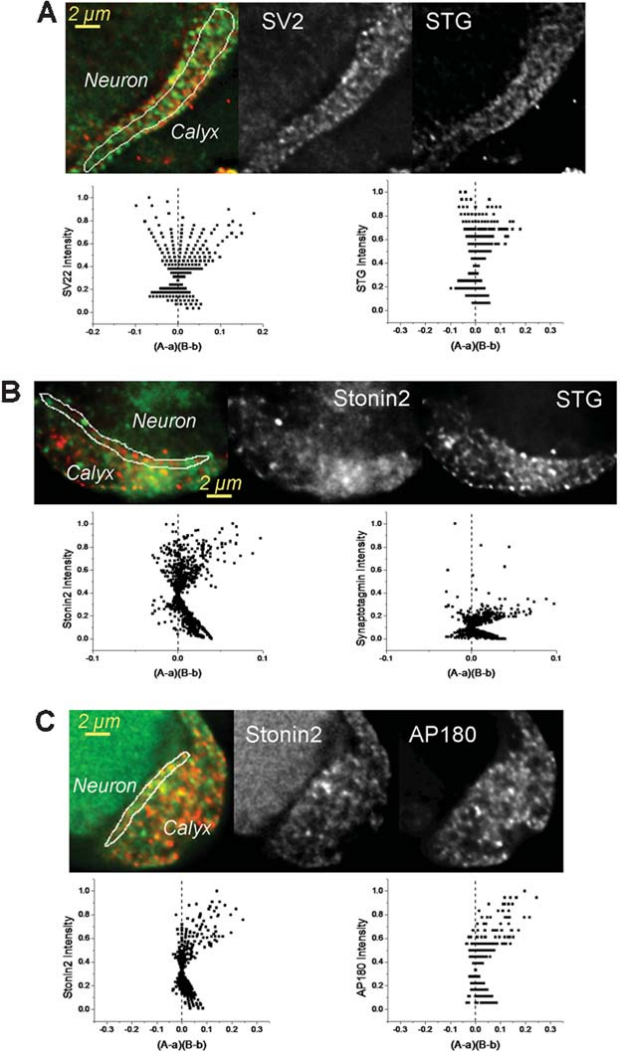
Localization of the SAE complex at the transmitter release face. Isolated chick ciliary ganglion calyx synapses were immunostained with antibodies against protein pairs. We used ICA plots to test for staining covariance (plot of normalized pixel intensity for each immunostain against the (*A-a*)(*B-b*) function, where *A* and *B* are the normalized pixel intensities for stain A and B and *a* and *b* are their respective means) in which a left skew indicates segregated staining, a symmetrical plot random staining and a right skew staining intensities that covary (see **Methods**). **A.** Calyx stained for secretory vesicle marker SV2 (***green, left panel***) and STG 1 (STG; ***red, left panel***) with ICA plots as labeled. The presynaptic calyx and postsynaptic neuron are indicated. The ***left panel*** is an overlay of the two black/white images. The ICA plots are for SV2 versus STG (***left panel***) and STG versus SV2 (***right panel***). **B.** As in **A** but staining for STG (***red, left panel, left ICA plot***) and the SAE component, stonin2 (***green, left panel, right ICA plot***). **C.** As in **A** but staining for Stonin2 (***green, left panel, left ICA plot***) and AP180 (***red, left panel, right ICA plot***).

It has been shown that STG is located both with synaptic vesicles and on the presynaptic transmitter release face membrane [20], [21]. We used the giant calyx-type synapse of the chick ciliary ganglion to carry out quantitative, high-resolution immunocytochemistry in order to localize the SAE complex. This synapse is ideal for such an analysis since we have previously demonstrated that it can be isolated intact on a cover-slip, and can be imaged at near light-limited optical resolution [13], [22]–[24]. Further, the large size of the presynaptic terminal permits a ready distinction of transmitter release face and intracellular regions of interest (ROIs). We first tested the hypothesis that the SAE complex is associated with SVs using the integral vesicular protein SV2 as a marker [13]. As expected, STG co-localized with the SVs in the intracellular region (***Fig. 7A***), a finding confirmed by ICA analysis with a positive mean ICQ value (0.12±0.02, N = 8; p_ = 0_<0.001). However, cytoplasmic SV2 staining did not covary with two other SAE members: stonin2 (*data not shown*, ICQ 0.02±0.01, N = 0; p_ = 0_>0.2) and AP180 (*data not shown*, ICQ 0.05±0.02, N = 8; p_ = 0_>0.05), arguing against an association of the complete SAE with SVs. At the transmitter release face STG covaried strongly with stonin2 (***Fig. 7B***; ICQ 0.143±0.030, N = 6; p_ = 0_<0.001), a key SAE component. Release face stonin2 also covaried with three other SAE components: AP180 (***Fig. 7C***; ICQ 0.18±0.02; N = 7; p_ = 0_<0.0001); S-/L-intersectin (*data not shown*, ICQ 0.143±0.020, N = 4; p_ = 0_<0.01) and H-clathrin (*data not shown*, ICQ 0.10±0.02; N = 5; p_ = 0_<0.01). These results localize SAE to the presynaptic transmitter release face STG pool but not to that with the synaptic vesicles.

## Discussion

We present a biochemical/immunocytochemical experimental method to derive a blueprint of an STG-containing multi-protein complex. Further, we demonstrate that this complex is associated with STG at the presynaptic transmitter release face.

This study reports a new strategy for the characterization of a multi-molecular protein complex based on a combination of co-immunoprecipitation, *FR* analysis and quantitative immunocytochemistry. Co-immunoprecipitation has been widely used to identify protein binding partners within a native biological tissue. As a stand-alone method it has, however, significant limitations. The method does not distinguish between moderately and strongly bound partners, false-positive rogue proteins can attach during antibody incubation and, with respect to larger protein complexes, little or no information is gained with respect to the binding relationship between a particular co-IP protein and the IP-protein. Strategies that are commonly used to improve the method include increasing the stringency of the co-precipitation conditions and biochemical cross-linking prior to tissue solubilization. The problem with the former is that while this will highlight proteins bound at high affinity this is not a guarantee of specificity and also, lower-affinity binding proteins may yet reflect important, if less stable, biological interactions. The cross-linking strategy can help with selectivity but the artificial strong binding precludes further analysis of complex internal organization. Our strategy was first, to use low-stringency conditions to co-precipitate a large population of IP-protein direct or indirect binding partners; second, use the ionic strength challenge to both provide an index of binding ‘strength’ and as a clue to multi-molecular complex organization; and third, ICA/ICQ analysis was used to test whether the putative protein partners covary *in situ*, as expected for two proteins that are parts of the same molecular complex.

The *FR* analysis method can generate an architectural blueprint of a multi-protein complex. The method is applicable to a group of proteins that co-precipitate and, hence, exhibit a high binding affinity relative to transient interactions such as with enzymes. It involves a number of simplifying assumptions and limitations which may significantly affect the accuracy of the ensuing blueprint. The method only distinguishes proteins that are susceptible to high-salt dissociation. Protein subsets that form highly stable structures within the complex, by, for example, covalent binding, would be expected to act as a single protein with a common *FR* value. We expect, however, that the method will work for complexes where the proteins are linked to several binding partners. Since protein shedding will only occur when the highest (electrostatic) affinity link is cleaved, the other binding links should simply not be detected and the resulting map will always reflect the strongest associations within the complex. It also assumes that the complex contains one example of each protein: more than one could generate a mixed *FR* value with an unclear interpretation. However, the method can be developed further by the use of a larger range of ionic strengths or salt treatment durations, permitting the identification of different binding relations. In addition, other methods could also be used to dissociate the complex such as pH, enzymatic cleavage or temperature.

One of the most interesting aspects of the *FR* analysis method is the ability to predict additional protein complex components. An opportunity to test this occurred when an antibody against stonin2 became available after the completion of most of this project (the models in ***Figure 4*** predate the stonin2 immunoprecipitation analysis). Not only did stonin2 co-precipitate with all complex members tested (***Fig. 5***), its measured *FR* values relative to STG, AP180, L-intersectin, S-intersectin and L-clathrin were remarkably consistent with values predicted from its putative location. Thus, our findings were consistent with the hypothesis that stonin2 bridges STG and intersectin in this complex. In addition, this experiment confirmed the predictive capability of the method. We anticipate that miniaturization and automation of these methods will allow the rapid generation of detailed blueprints for small to mid-sized protein complexes.

The blueprint for the SAE describes an AP180/*X*/STG/stonin 2/intersectin/epsin core (where *X* is unknown and epsin is hypothesized), together with peripheral AP2 adaptor, H-/L-clathrin coat and dynamin scission proteins (***Fig. 6***). We can be more confident about the locations of the core proteins than the peripheral ones as the *FR* coordinates are less reliable when both values are low. Smaller fragments of a similar complex have been reported previously: one of the more interesting members of the complex, the binding of stonin2 to STG has been recently noted [25] and these proteins also associate with AP2 [19]. In a separate study (Khanna et al., *unpublished*) we have noted a different endocytotic protein complex that is associated with transmitter release sites, as identified by the calcium channel. However, that entity and the SAE differ most notably by the absence of AP2 from the former. The SAE is consistent with previous reports on endocytotic protein binding [5], [16], [19], [25]–[35] and may reflect the parent complex explored in those reports.

The presence of STG in the SAE suggested that this complex was associated with SVs. However, an alternative location is implicated by the involvement of STG in SV recovery [19], [36], [37]. Our observation that the SAE complex members do not co-localize with SV markers within the terminal together with previous biochemical analyses that failed to identify clathrin coat proteins with purified SVs [38] argue against an association with SVs. Recent studies have identified a significant pool of STG in the presynaptic surface membrane that exchanges with the SV pool during exocytosis [20], [21]. Our immunostaining analysis suggests that the SAE is associated with this pool. Very recent findings by Diril et al. [19] suggest that stonin2 acts together with AP2 as a sorting adaptor for STG in clathrin-dependent internalization. The SAE complex described here supports and expands on this hypothesis by confirming a close association between stonin2, STG and AP2 while describing a specific multi-molecular complex that includes other adaptor proteins in addition to clathrin coat proteins. Since this complex must be abundant in the presynaptic terminals, we speculate that it may act not only as a sorting adaptor, but also as a seed entity to initiate clathrin coat formation. If, as suggested, STG serves as the Ca sensor and also plays a key role in exocytosis, using this protein as the key endocytosis adaptor would ensure that it is always included in the newly formed SV.

## Methods

### Antibodies

Dilutions and source of antibodies are shown in Table 1. All antibodies were tested for specificity by Western blot (*data not shown*).

**Table 1:**
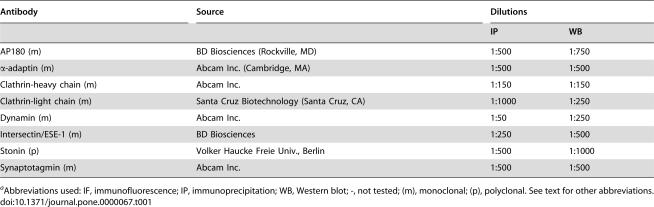
(Methods). Antibodies used in this study.

Antibody	Source	Dilutions	
		IP	WB
AP180 (m)	BD Biosciences (Rockville, MD)	1∶500	1∶750
*α*-adaptin (m)	Abcam Inc. (Cambridge, MA)	1∶500	1∶500
Clathrin-heavy chain (m)	Abcam Inc.	1∶150	1∶150
Clathrin-light chain (m)	Santa Cruz Biotechnology (Santa Cruz, CA)	1∶1000	1∶250
Dynamin (m)	Abcam Inc.	1∶50	1∶250
Intersectin/ESE-1 (m)	BD Biosciences	1∶250	1∶500
Stonin (p)	Volker Haucke Freie Univ., Berlin	1∶500	1∶1000
Synaptotagmin (m)	Abcam Inc.	1∶500	1∶500

a Abbreviations used: IF, immunofluorescence; IP, immunoprecipitation; WB, Western blot; -, not tested; (m), monoclonal; (p), polyclonal. See text for other abbreviations.

### Synaptosome preparation

Synaptosomes were prepared as described [22]. Briefly, brains from neonatal (2–10 days old) Sprague Dawley rats were dissected into 10 volumes of ice-cold homogenization buffer (0.32 M sucrose, 10 mM HEPES pH 7.4, 2 mM EDTA, supplemented with protease inhibitors) and homogenized using 10–15 strokes of a glass Teflon hand-held homogenizer. The homogenate was then spun for 15 minutes at 1000× g 4°C and the supernatant was spun for 45 minutes at 200,000× g 4°C. The res-suspended pellet was spun at 200,000× g for 45 minutes. This second pellet (P2) was re-suspended in HEPES lysis buffer (50mM HEPES pH 7.4, 2 mM EDTA plus protease inhibitors), layered onto 4 ml of 1.2M sucrose and centrifuged at 230,000× g (4°C) for 30 minutes. The gradient interphase was diluted in 7–8 mls of ice-cold HEPES-buffered sucrose (0.32 M sucrose, 4 mM HEPES, pH 7.4) and layered onto 4 ml of 0.8 M sucrose and re-centrifuged at 230,000× g for 15 minutes (4°C). The pellet was re-homogenized in modified RIPA buffer; filtered through a 0.22 µm syringe filter and stored at −80°C.

### Immunoprecipitation and Western blotting [22]

Synaptosomes were pre-cleared by a 1 hr incubation with 20 µl of a 50% slurry of protein A beads (Pierce, Rockford, IL) and incubated overnight with primary or control antibodies. Antibody-captured complexes were recovered with fresh protein A or protein A/G (for rabbit or mouse antibodies, respectively) agarose beads (20 µl original bead slurry) by incubation with lysate-antibody mixture at 4°C for 2 hr and were then washed 3 times. Protein samples were boiled in Laemmli sample buffer for 5 minutes prior to fractionation on 5%, 7.5%, 10% or 4–15% separating gels with 4% stacking gels. Proteins were transferred to PVDF membranes (Invitrogen) for immunoblotting or stained with Amido black (BioRad) to monitor transfer efficiency. Western blots were performed using standard procedures (Khanna et al., 2006). The membranes were blocked for 1 hr in 5% skim milk powder in TBST (25 mM Tris-Cl, pH 8.0, 125 mM NaCl, 0.1% Polyoxyethylene Sorbitan Monolaurate (Tween-20)) at room temperature. Primary antibody incubations were for 2 hr at room temperature or overnight at 4°C. After secondary antibody treatment (goat anti-rabbit or anti-mouse IgG horseradish peroxidase, Stressgen; 1∶5000), blots were washed extensively in TBST and probed with Enhanced Chemiluminescence reagent (NEN Life Science) before exposure to photographic film. Films were exposed for varying periods to ensure a non-saturated example suitable for quantitative analysis. Control gels with titrated protein concentrations demonstrated the near-linear range of the film (***Supporting Fig. S1A, B***). Protein bands were scanned (Canoscan LiDE 30, Canon, Mississauga, Ontario) at an image quality of 600 dpi, digitized and quantified using Un-Scan-It gel V6.1 scanning software (Silk Scientific Inc., Orem, UT).

### High salt treatment of immunoprecipitates

Immunoprecipitation was carried out as above. After the triple wash step the beads were split into three equal aliquots and were incubated for 15 min on ice in RIPA buffer with 150, 650 or 1150 mM total salt, respectively, vortexing once. The immunoprecipitate-carrying beads were spun briefly and the supernatant discarded. The beads were boiled in Laemmle sample buffer for 5 minutes and liberated proteins were analyzed by electrophoresis and Western blot, as above. Each trial consisted of three samples, two at high salt (650 and 1150 mM) and a matched 150 mM salt control.

### Database mining

On-line database (Biomolecular Interaction Network Database (BIND; http://www.bind.ca) and Protein Protein Interaction Database (PPID; H. Husi; http://www.anc.ed.ac.uk/mscs/PPID/) were used to screen but we relied on cited literature.

### Chick ciliary ganglion calyx synapse preparation

This has been described in detail [13], [23], [39], [40]. After trituration of the ganglia the cells/terminal preparation was plated at 37°C in a standard cell incubator for 45 minutes prior to fixation and staining.

### Immunostaining, Microscopy and Iterative deconvolution deblurring

This has been described in detail [13], [23]. Microscopy techniques were as described [13]. Regions of interest (ROIs) were identified by eye from the sampled and neighboring optical sections.

### Intensity Correlation Analysis (ICA)/Intensity Correlation Quotient (ICQ)

This analysis has been described in detail [13]. Basically, for the ICA we calculated the function (*Ai*–*a*)(*Bi*–*b*), where *a* and *b* are the means of each pixel staining pair intensity values *Ai* and *Bi*. *Ai* or *Bi* was graphed in separate scatter plots against their respective (*Ai*–*a*)(*Bi*–*b*) value. Distributions that skew to the right reflect dependent staining patterns (where the two pixel staining intensity values vary in synchrony), ones that are symmetrical about the 0 axis indicate random staining, while those that skew to the left reflect independent staining patterns, where the pixel staining intensity values vary inversely. Note that the analysis can be carried out for each stain separately so that a dependence of stain *A* on *B* but a lack of dependence of *B* on *A* can be identified and, further, that the plots permit detection of complex or mixed staining relations. The intensity correlation quotient (ICQ) reflects the ratio of the number of positive (*Ai*–*a*)(*Bi*–*b*) values to the total number of pixels in the ROI, corrected to a −0.5 (independent staining) to +0.5 (dependent staining) range by subtracting 0.5. The ICQ provides a single value indication that can be used for statistical comparison. Typically, with *N*>6 ROIs, a mean ICQ value of −.05 to +.05 indicates random staining, +.05 to +.10, indicates a moderate covariance and >.1 a strong covariance. The mean ICQ was tested for equaling zero (p_ = 0_) using a standard Students t-test. ICA/ICQ analysis was carried out by means of an automated graphic plugin ([22], Image Correlation Analysis for the public domain image analysis software ImageJ (Wayne Rasband; Research Services Branch, National Institutes of Health, Bethesda MD using the package and plugin available at the Wright Cellular Imaging Facility, Toronto Western Research Institute, UHN.

### Image presentation

Images were cut, background was subtracted (using an area outside the cell), overlays were created and brightness/contrast was adjusted with ImageJ and PowerPoint software. No non-linear or partial-image adjustments were made.

## Supporting Information

Figure S1 Calibration of protein recovery and reciprocity of immunoprecipitations. (A) Western blot analysis of a gel loaded with various amounts of rat brain synaptosomes at concentration between 1.25 and 30 mg and immunoblotted for synaptotagmin (STG). (B) Plot of band intensity against amount of protein loaded. Following exposure to film and scanning, bands were quantified using Un-SCAN-IT gel 6.1. The data were fit by a straight line. Detection of proteins bands was linear with respect to the amount of protein. (C) Comparison of fractional recovery (FR) values for protein pairs using one or other as the immunoprecipitated (IP)-protein (as indicated on y-axis). The rows show the FR for the same protein pair, immunoprecipitating protein A and probing for protein B or vice versa (as labeled, Fig. S1C). Note the similar FR values in either direction, as would be expected if these reflect the same molecular interactions.
(0.08 MB TIF)

Figure S2 Fractional recovery (FR) series congruence analysis. Each plot compares two FR sequences for congruence and, hence, origin from the same protein complex. The filled symbols show the set of mean±SE FR sequence for a test IP-protein (replotted from Fig. 2A-E). The open symbols show the FR sequence for a second IP-protein adjusted for the differences in the FR values of the two IP-proteins. Note that most of the transformed IP-protein predictions are within 2SE of the test protein, supporting their origin from the same protein complex. The test IP-protein/transformed IP-protein pairs are: A. AP180/H-clathrin, B. AP180/synaptotagmin, C. L-clathrin/synaptotagmin, and D. S-intersectin/synaptotagmin.
(0.07 MB TIF)

## References

[1] Gentile L, Stanley EF (2005). A unified model of presynaptic release site gating by calcium channel domains.. Eur J Neurosci.

[2] Murthy VN, De Camilli P (2003). Cell biology of the presynaptic terminal.. Annu Rev Neurosci.

[3] Augustine GJ, Morgan JR, Villalba-Galea CA, Jin S, Prasad K (2006). Clathrin and synaptic vesicle endocytosis: studies at the squid giant synapse.. Biochem Soc Trans.

[4] Mousavi SA, Malerod L, Berg T, Kjeken R (2004). Clathrin-dependent endocytosis.. Biochem J.

[5] Lafer EM (2002). Clathrin-protein interactions.. Traffic.

[6] Slepnev VI, De Camilli P (2000). Accessory factors in clathrin-dependent synaptic vesicle endocytosis.. Nat Rev Neurosci.

[7] Xiong X, Zhou KM, Wu ZX, Xu T (2006). Silence of synaptotagmin I in INS-1 cells inhibits fast exocytosis and fast endocytosis.. Biochem Biophys Res Commun.

[8] Jorgensen EM, Hartwieg E, Schuske K, Nonet ML, Jin YS (1995). Defective recycling of synaptic vesicles in synaptotagmin mutants of *Caenorhabditis elegans*.. Nature(Lond ).

[9] Littleton JT, Bai J, Vyas B, Desai R, Baltus AE (2001). synaptotagmin mutants reveal essential functions for the C2B domain in Ca2+-triggered fusion and recycling of synaptic vesicles in vivo.. J Neurosci.

[10] Nicholson-Tomishima K, Ryan TA (2004). Kinetic efficiency of endocytosis at mammalian CNS synapses requires synaptotagmin I.. Proc Natl Acad Sci U S A.

[11] Poskanzer KE, Fetter RD, Davis GW (2006). Discrete residues in the c(2)b domain of synaptotagmin I independently specify endocytic rate and synaptic vesicle size.. Neuron.

[12] Willig KI, Rizzoli SO, Westphal V, Jahn R, Hell SW (2006). STED microscopy reveals that synaptotagmin remains clustered after synaptic vesicle exocytosis.. Nature(Lond ).

[13] Li Q, Lau A, Morris TJ, Guo L, Stanley EF (2004). A syntaxin 1, Galpha(o), and N-type calcium channel complex at a presynaptic nerve terminal: analysis by quantitative immunocolocalization.. J Neurosci.

[14] Yamabhai M, Hoffman NG, Hardison NL, McPherson PS, Castagnoli L (1998). Intersectin, a novel adaptor protein with two Eps15 homology and five Src homology 3 domains.. J Biol Chem.

[15] Kelly LE, Phillips AM (2005). Molecular and genetic characterization of the interactions between the Drosophila stoned-B protein and DAP-160 (intersectin).. Biochem J.

[16] Martina JA, Bonangelino CJ, Aguilar RC, Bonifacino JS (2001). Stonin 2: an adaptor-like protein that interacts with components of the endocytic machinery.. J Cell Biol.

[17] Montesinos ML, Castellano-Munoz M, Garcia-Junco-Clemente P, Fernandez-Chacon R (2005). Recycling and EH domain proteins at the synapse.. Brain Res Brain Res Rev.

[18] Drake MT, Downs MA, Traub LM (2000). Epsin binds to clathrin by associating directly with the clathrin-terminal domain. Evidence for cooperative binding through two discrete sites.. J Biol Chem.

[19] Diril MK, Wienisch M, Jung N, Klingauf J, Haucke V (2006). Stonin 2 is an AP-2-dependent endocytic sorting adaptor for synaptotagmin internalization and recycling.. Dev Cell.

[20] Fernandez-Alfonso T, Kwan R, Ryan TA (2006). Synaptic Vesicles Interchange Their Membrane Proteins with a Large Surface Reservoir during Recycling.. Neuron.

[21] Wienisch M, Klingauf J (2006). Vesicular proteins exocytosed and subsequently retrieved by compensatory endocytosis are nonidentical.. Nat Neurosci.

[22] Khanna R, Li Q, Sun L, Collins TJ, Stanley EF (2006). N type Ca(2+) channels and RIM scaffold protein covary at the presynaptic transmitter release face but are components of independent protein complexes.. Neuroscience.

[23] Mirotznik RR, Zheng X, Stanley EF (2000). G-Protein types involved in calcium channel inhibition at a presynaptic nerve terminal.. J Neurosci.

[24] Stanley EF, Mirotznik RR (1997). Cleavage of syntaxin prevents G-protein regulation of presynaptic calcium channels.. Nature(Lond ).

[25] Walther K, Diril MK, Jung N, Haucke V (2004). Functional dissection of the interactions of stonin 2 with the adaptor complex AP-2 and synaptotagmin.. Proc Natl Acad Sci U S A.

[26] Tebar F, Sorkina T, Sorkin A, Ericsson M, Kirchhausen T (1996). Eps15 is a component of clathrin-coated pits and vesicles and is located at the rim of coated pits.. J Biol Chem.

[27] Okamoto M, Schoch S, Sudhof TC (1999). EHSH1/intersectin, a protein that contains EH and SH3 domains and binds to dynamin and SNAP-25. A protein connection between exocytosis and endocytosis?. J Biol Chem.

[28] Keen JH, Chestnut MH, Beck KA (1987). The clathrin coat assembly polypeptide complex. Autophosphorylation and assembly activities.. J Biol Chem.

[29] Roos J, Kelly RB (1998). Dap160, a neural-specific Eps15 homology and multiple SH3 domain-containing protein that interacts with Drosophila dynamin.. J Biol Chem.

[30] Marie B, Sweeney ST, Poskanzer KE, Roos J, Kelly RB (2004). Dap160/intersectin scaffolds the periactive zone to achieve high-fidelity endocytosis and normal synaptic growth.. Neuron.

[31] Hussain NK, Yamabhai M, Ramjaun AR, Guy AM, Baranes D (1999). Splice variants of intersectin are components of the endocytic machinery in neurons and nonneuronal cells.. J Biol Chem.

[32] Blondeau F, Ritter B, Allaire PD, Wasiak S, Girard M (2004). Tandem MS analysis of brain clathrin-coated vesicles reveals their critical involvement in synaptic vesicle recycling.. Proc Natl Acad Sci U S A.

[33] Koh TW, Verstreken P, Bellen HJ (2004). Dap160/intersectin acts as a stabilizing scaffold required for synaptic development and vesicle endocytosis.. Neuron.

[34] Walther K, Krauss M, Diril MK, Lemke S, Ricotta D (2001). Human stoned B interacts with AP-2 and synaptotagmin and facilitates clathrin-coated vesicle uncoating.. EMBO Rep.

[35] Yao PJ, Petralia RS, Bushlin I, Wang Y, Furukawa K (2005). Synaptic distribution of the endocytic accessory proteins AP180 and CALM.. J Comp Neurol.

[36] Zhang JZ, Davletov BA, Südhof TC, Anderson RGW (1994). Synaptotagmin I is a high affinity receptor for clathrin AP-2: Implications for membrane recycling.. Cell.

[37] von Poser C, Zhang JZ, Mineo C, Ding W, Ying Y (2000). Synaptotagmin regulation of coated pit assembly.. J Biol Chem.

[38] Maycox PR, Link E, Reetz A, Morris SA, Jahn R (1992). Clathrin-coated vesicles in nervous tissue are involved primarily in synaptic vesicle recycling.. J Cell Biol.

[39] Stanley EF (1991). Single calcium channels on a cholinergic presynaptic nerve terminal.. Neuron.

[40] Stanley EF, Goping G (1991). Characterization of a calcium current in a vertebrate cholinergic presynaptic nerve terminal.. J Neurosci.

