# Supplementary figures and images for: ‘Fractional Recovery’ Analysis of a Presynaptic Synaptotagmin 1-Anchored Endocytic Protein Complex
Source: PLoS One. 2006 Dec 20;1(1):e67. doi: 10.1371/journal.pone.0000067 (PMC1762330; doi:10.1371/journal.pone.0000067)

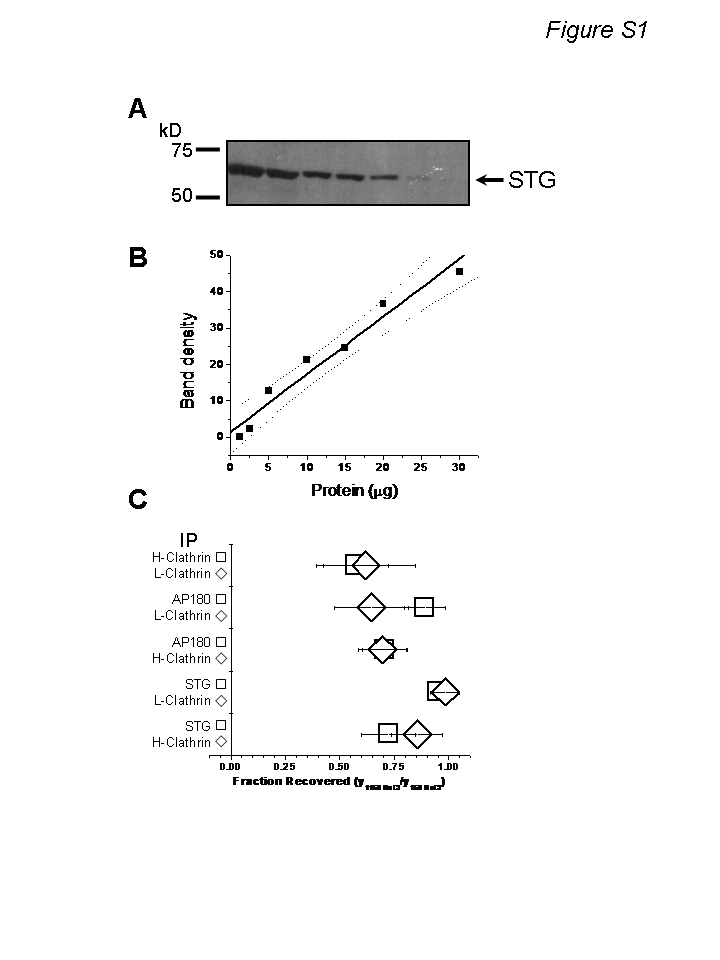

Supplement: Figure S1 — Calibration of protein recovery and reciprocity of immunoprecipitations. (A) Western blot analysis of a gel loaded with various amounts of rat brain synaptosomes at concentration between 1.25 and 30 mg and immunoblotted for synaptotagmin (STG). (B) Plot of band intensity against amount of protein loaded. Following exposure to film and scanning, bands were quantified using Un-SCAN-IT gel 6.1. The data were fit by a straight line. Detection of proteins bands was linear with respect to the amount of protein. (C) Comparison of fractional recovery (FR) values for protein pairs using one or other as the immunoprecipitated (IP)-protein (as indicated on y-axis). The rows show the FR for the same protein pair, immunoprecipitating protein A and probing for protein B or vice versa (as labeled, Fig. S1C). Note the similar FR values in either direction, as would be expected if these reflect the same molecular interactions. (0.08 MB TIF) [file pone.0000067.s001.tif]

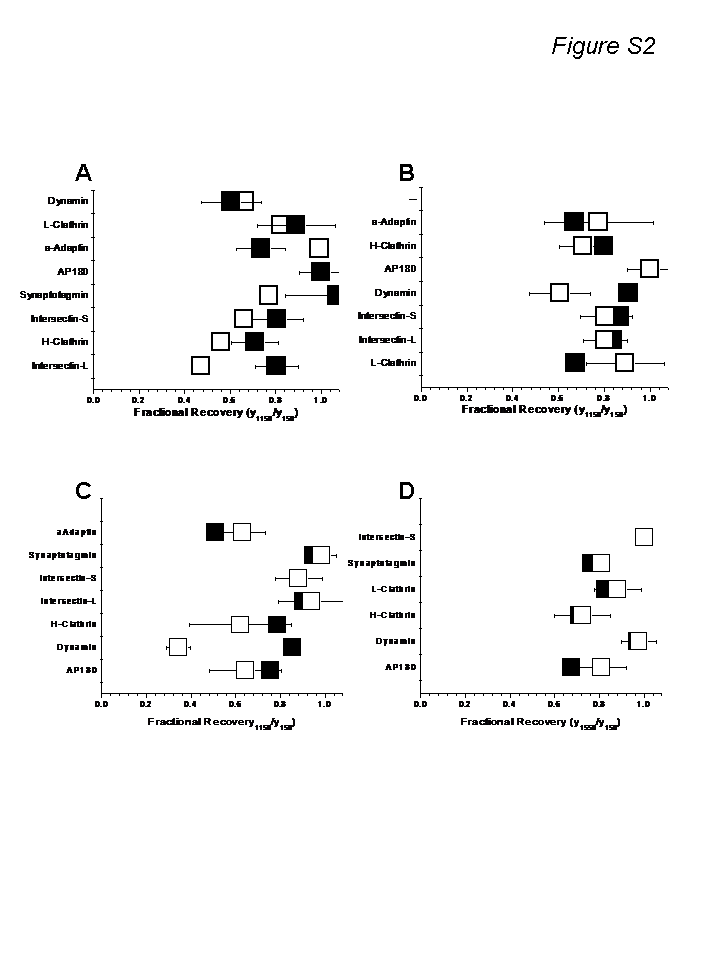

Supplement: Figure S2 — Fractional recovery (FR) series congruence analysis. Each plot compares two FR sequences for congruence and, hence, origin from the same protein complex. The filled symbols show the set of mean±SE FR sequence for a test IP-protein (replotted from Fig. 2A-E). The open symbols show the FR sequence for a second IP-protein adjusted for the differences in the FR values of the two IP-proteins. Note that most of the transformed IP-protein predictions are within 2SE of the test protein, supporting their origin from the same protein complex. The test IP-protein/transformed IP-protein pairs are: A. AP180/H-clathrin, B. AP180/synaptotagmin, C. L-clathrin/synaptotagmin, and D. S-intersectin/synaptotagmin. (0.07 MB TIF) [file pone.0000067.s002.tif]
